# Generation of BAC Transgenic Tadpoles Enabling Live Imaging of Motoneurons by Using the Urotensin II-Related Peptide (*ust2b*) Gene as a Driver

**DOI:** 10.1371/journal.pone.0117370

**Published:** 2015-02-06

**Authors:** Marion Bougerol, Frédéric Auradé, François M. Lambert, Didier Le Ray, Denis Combes, Muriel Thoby-Brisson, Frédéric Relaix, Nicolas Pollet, Hervé Tostivint

**Affiliations:** 1 Evolution des Régulations Endocriniennes, UMR 7221 CNRS, Muséum National d’Histoire Naturelle, Paris, France; 2 UPMC/INSERM UMRS 974, CNRS FRE 3617, AIM, Paris, France; 3 INSERM, Avenir Team, Pitié-Salpêtrière, Paris, France; 4 UMR 5287 CNRS, Université de Bordeaux, INCIA, Bordeaux, France; 5 Institute of Systems and Synthetic Biology, CNRS, Université d’Evry Val d’Essonne, Evry, France; Inserm, FRANCE

## Abstract

*Xenopus* is an excellent tetrapod model for studying normal and pathological motoneuron ontogeny due to its developmental morpho-physiological advantages. In mammals, the urotensin II-related peptide (*UTS2B*) gene is primarily expressed in motoneurons of the brainstem and the spinal cord. Here, we show that this expression pattern was conserved in *Xenopus* and established during the early embryonic development, starting at the early tailbud stage. In late tadpole stage, *uts2b* mRNA was detected both in the hindbrain and in the spinal cord. Spinal *uts2b^+^* cells were identified as axial motoneurons. In adult, however, the *uts2b* expression was only detected in the hindbrain. We assessed the ability of the *uts2b* promoter to drive the expression of a fluorescent reporter in motoneurons by recombineering a green fluorescent protein (GFP) into a bacterial artificial chromosome (BAC) clone containing the entire *X. tropicalis uts2b* locus. After injection of this construction in one-cell stage embryos, a transient GFP expression was observed in the spinal cord of about a quarter of the resulting animals from the early tailbud stage and up to juveniles. The GFP expression pattern was globally consistent with that of the endogenous *uts2b* in the spinal cord but no fluorescence was observed in the brainstem. A combination of histological and electrophysiological approaches was employed to further characterize the GFP^+^ cells in the larvae. More than 98% of the GFP^+^ cells expressed choline acetyltransferase, while their projections were co-localized with α-bungarotoxin labeling. When tail myotomes were injected with rhodamine dextran amine crystals, numerous double-stained GFP^+^ cells were observed. In addition, intracellular electrophysiological recordings of GFP^+^ neurons revealed locomotion-related rhythmic discharge patterns during fictive swimming. Taken together our results provide evidence that *uts2b* is an appropriate driver to express reporter genes in larval motoneurons of the *Xenopus* spinal cord.

## Introduction

Generation of transgenic lines with promoter-specific fluorescent reporter proteins has significantly advanced neurobiological research by enabling the visualization of neuronal subsets *in vivo*. For genetic engineering of such animals, mice have long been the most frequently used experimental vertebrate model [1]. However, organisms such as zebrafish and *Xenopus* have recently emerged as alternative systems and are now being widely exploited to study human neurological diseases as well as to screen for potential therapeutics [2–5]. Several features of these species make them particularly amenable for neurodevelopmental and neurophysiological investigations including their rapid development rate as free-living larvae, their transparency allowing easy visualization of internal structures and cells in live animals, and the general organization of their central nervous system (CNS) which is quite similar to other vertebrate species, including human. Moreover, high-quality and well annotated sequenced genomes exist for both species [6, 7] and many readily available genome editing technologies have been successfully adapted to them [8–11]. *Xenopus*, as a tetrapod, offers the added advantage of being much more closely related to mammals than the zebrafish. This proximity is especially important when studying the neural control of processes that have no genuine equivalent in fish, such as legged terrestrial locomotion [12].

For engineering useful transgenic reporter genes, a crucial step is to choose the correct sequences which will drive the fluorescent reporter in the neuronal cell type of interest. In the case of motoneurons, the regulatory sequences of several genes encoding transcription factors involved in motoneuron specification, such as *HB9* (also named *Mnx1*), *ISLET-1* (*ISL1*) and *OLIG2*, were shown to be particularly well suited, especially in zebrafish [13–15]. In *Xenopus*, generation of transgenic motoneuronal reporter lines using *hb9* as a driver is currently in progress [16, NP, unpublished results]. Despite their great interest, all these genes have the disadvantage of also being expressed in other neurons than motoneurons, not only in the brain but also in the spinal cord. Typically, *olig2* is expressed within the progenitor domain that gives rise both to motoneurons and oligodendrocytes [17]. Likewise, *isl1* and *hb9* are expressed in motoneurons as well as in some sensory neurons and/or interneurons [18, 19].

Urotensin II (UTS2) and urotensin II-related peptide, also known as urotensin 2B (UTS2B), are two structurally and phylogenetically related neuropeptides [20, 21] that, at least in tetrapods, are mainly expressed in motoneurons of the brainstem and the spinal cord [22–29]. UTS2 and UTS2B have been shown to be of biological importance, playing an important role in the regulation of behavior, neuroendocrine activities, and central and peripheral control of blood pressure and heart rate [30–33]. In mammals, all these effects are mediated by only one receptor known as UT, which is the reason why the proper effects of each peptide are often difficult to discriminate [21]. Even though the functional significance of their motoneuronal expression is currently poorly understood, UTS2 and UTS2B can be considered as two interesting putative spinal motoneuronal markers.

Here, our aim was to test the ability of the *uts2b* promoter to drive the expression of the green fluorescent protein (GFP) specifically in motoneurons. Up to now, functional studies on the *uts2b* promoter have never been carried out in *Xenopus*. Moreover, sequence analyses did not reveal any conserved motif which would be involved in the motoneuronal expression of the *uts2b* gene. One significant limitation in generating transgenic reporter genes is that the regulatory elements that control the gene transcription can be scattered over large regions, therefore making their identification quite difficult and time consuming, especially when they are not conserved among species. This limitation can be overcome by placing the reporter gene into large-insert clones such as bacterial artificial chromosomes (BAC) that are thought to contain all the regulatory sequences driving its appropriate expression [34]. For this purpose, we recombined a *GFP* reporter gene into a BAC clone containing the entire *X. tropicalis uts2b* locus and employed a recently reported method of BAC injection into *X. laevis* embryo [35]. In this method, the transgene construct is delivered as a large circular DNA which does not promote its genomic integration. Yet, such large DNA constructs can be replicated and then maintained as episomes over cell divisions. It is therefore believed that the reporter gene can be only transiently transcribed. Combining various histological and electrophysiological approaches, we observed that up to a quarter of injected embryos expressed *GFP* in a manner consistent with endogenous *uts2b* expression in spinal cord, *i.e.* almost exclusively (up to 98%) in motoneurons. Thus, our results showed that *uts2b* is an appropriate driver to express reporter genes in the *Xenopus* spinal motoneurons.

## Materials and Methods

### Animals

Studies were performed on the South African clawed toad *X. laevis* obtained from the Centre de Ressources Biologiques Xénope in France (CNRS and University of Rennes 1; http://xenopus.univ-rennes1.fr/) and maintained at 20–22°C in aquaria exposed to a 12:12 h light/dark cycle. Embryos were obtained by breeding adult frogs after injection of human chorionic gonadotropin (hCG) (Chorulon) (400 units/female and 200 units/male) or after *in vitro* fertilization as described in [36]. Tadpoles were raised in Marc’s Modified Ringers (MMR) solution (100 mM NaCl, 2 mM KCl, 1 mM MgCl_2_, 2 mM CaCl_2_, 5 mM HEPES, pH 7.5). Animals were sorted according to the developmental stages outlined by Nieuwkoop and Faber [37]. All procedures were carried out in accordance with and approved by the local ethics committees: the Comité d’Ethique Cuvier du Museum National d’Histoire Naturelle (protocols # 68–019 to HT) and the Comité d’Ethique de Bordeaux en Expérimentation Animale (protocols # 3301100012-A to DLR).

### Construction of the uts2b-EGFP BAC transgene

**Fig 1:**
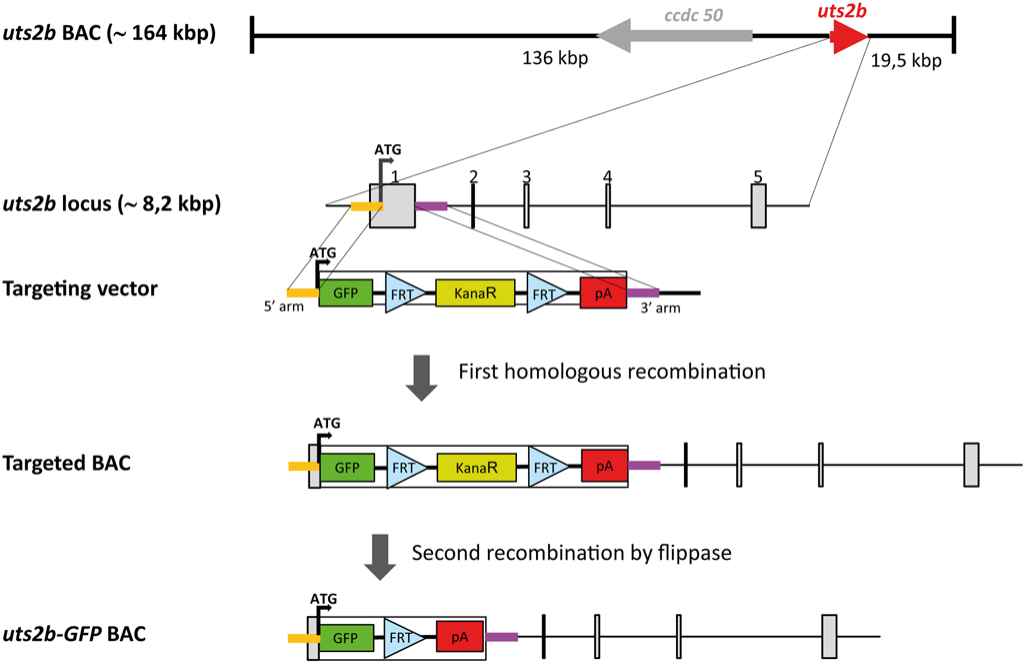
Strategy for constructing the recombinant *uts2b* BAC containing *uts2b*-EGFP expression cassette. The *X. tropicalis uts2b* BAC clone, ALNOAAA14YB24, contains approximately 164 kbp genomic DNA. The *uts2b* gene is approximately 8.2 kbp long as represented by a red arrow, and it comprises 5 exons. The targeting vector was designed to contain two *uts2b* genomic DNAs on its either side (denoted 5’- and 3’-arms), that allows a specific homologous recombination between the targeting vector and the *uts2b* BAC DNA at the genomic region surrounding the first exon. Homology arms for the first exon of *uts2b* were ligated to both ends of an EGFP.FRT-Promotor Bacterial Minimal.kanamycin-FRT.2pA expression cassette. Through the first homologous recombination, the expression cassette was inserted in place of the first *uts2b* exon in the *uts2b* BAC clone. Then, the kanamycin resistance cassette was selectively eliminated by expressing Flipase, leading to the *uts2b*-EGFP BAC clone containing only the EGFP cDNA expression cassette under control of the *uts2b* promoter. Finally, note that the *ccdc50* gene, located close to the *uts2b* locus, was deleted from the *uts2b* BAC to avoid possible artifacts due to its overexpression (not shown).

The BAC clone ALN0AAA14YB24 was obtained from a custom BAC library from *X. tropicalis* Adiopodoume strain genomic DNA prepared in the pECBAC1 vector (NP, unpublished). All BAC clones from this library were end-sequenced and mapped to the *X. tropicalis* genome sequence version 7.1. We crossed the mapping information of the *uts2b* gene obtained from Xenbase (scaffold_5:5523406–5531650) and identified three BAC clones overlapping this genomic region. The BAC ALN0AAA14YB24 was the most interesting because both of its end sequences that could be unambiguously aligned on the genome. Thus ALN0AAA14YB24 contained theoretically ∼164 kilo-base pairs (kbp) of *X. tropicalis* genomic DNA including the whole *uts2b* transcription unit (scaffold_5:5387394–5551119, Genbank: JS994624.1 and JS988846.1). We then estimated ALN0AAA14YB24 BAC DNA insert size by pulsed-field gel electrophoresis after a NotI restriction digest. A single NotI restriction fragment of around 180 +/− 10 kbp was obtained, in agreement with the theoretical expectation. Thus this BAC should contain sequences spanning ∼136 kbp upstream and ∼19.5 kbp downstream of the *uts2b* gene (Fig. 1). On account of its features, it is expected that the ALN0AAA14YB24 clone contains most, if not all, regulatory elements necessary to recapitulate the endogenous expression of *uts2b in vivo*.

The open reading frame of the *uts2b* gene was replaced by an enhanced GFP (EGFP).FRT- kanamycin-FRT.2polyA cassette by recombineering-based cloning strategy [38]. Two homology arms of roughly 550 bp were chosen and amplified by PCR (see S1 Table for the primer sequences) and assembled in pSKT with NcoI in the middle so that the ATG contained in the NcoI site corresponds to *uts2b* ATG with minor alterations of the 5’UTR (2 bases substitution upstream of the ATG). The expression cassette was inserted between the recombination arms in the NcoI pivotal site by in-fusion PCR using the In-Fusion HD Cloning kit (Clontech; see S1 Table for the primer sequences) resulting in the positioning of the EGFP ATG in place of the *uts2b* ATG. BAC ALN0AAA14YB24 DNA was electroporated into recombineering strain SW105 [39]. BAC DNA from resulting clones was analyzed for BAC integrity by restriction profile using EcoRI+NotI and EcoRV+NotI. A good clone was then electroporated with the recombination cassette. Kanamycin resistant recombinant clones were analyzed by restriction profile for proper cassette integration. A clone satisfying the criteria was then grown on arabinose to induce the Flp recombinase which led to excision of the kanamycin cassette.

In addition to *uts2b*, the ALN0AAA14YB24 clone contains the complete locus of *ccdc50* which encodes for an effector of EGF-mediated cell signaling shown to be required for survival of some cell types [40–42]. To avoid possible artifacts due to its overexpression, *ccdc50* was inactivated in the reporter BAC through the insertion in the coding sequence of exon1 of stop codons on the 3 reading frames and multimerized polyA signals, further resulting in the destruction of the exon1 splice donor site (see S1 Table for the primer sequences used).

### Generation of transgenic animals

BAC DNA was purified with Nucleobond BAC100 (Macherey-Nagel, Düren, Germany) and dialyzed against microinjection buffer (10 mM Tris-HCl, pH 7.5, 0.1 mM EDTA, 30 µM spermine, 70 µM spermidine, 100 mM NaCl) and stored at 4°C [35, 43]. On the day of injections, DNA was diluted to 100 ng/µl in sterile water, and further diluted in sperm dilution buffer (250 mM sucrose, 75 mM KCl, 0.5 mM spermidine trihydrochloride, 0.2 mM spermine tetrahydrochloride, pH 7.3–7.5) to the appropriate concentration (5 pg/nl) for injections. Embryos were injected using a Microinjector 5242 (Eppendorf, Germany) at the one-cell stage with 4 nL of the solution containing the BAC DNA.

### Synthesis of the riboprobes for *in situ* hybridization

To generate the *uts2b* probe, a PCR fragment of 638 bp was amplified from adult *X. laevis* brain and spinal cord RACE-ready cDNA (see S1 Table for the primer sequences) then subcloned into pGEM-T easy (Promega, Charbonnières, France). Sense and antisense digoxigenin (Dig)-labeled probes were synthesized from the linearized plasmid with the RNA polymerases T7 and Sp6, respectively, using the RNA Labeling Kit (Roche Diagnostics, Mannheim, Germany).

### *In situ* hybridization

*In situ* hybridization was performed as previously described [44, 45] on whole tadpoles (up to stage 40), dissected CNS (from stages 48 to 60) or CNS sections (18 µm). To facilitate observations, whole CNS preparations were longitudinally incised with fine-tip scissors and opened up like a book. To reveal *uts2b* expression, probes were detected with anti-Dig antibodies conjugated to alkaline phosphatase followed by a chromogenic reaction using a solution of BM Purple (Roche Diagnostics) or Fast Red (Roche Diagnostics) as substrates. Alternatively, in particular to ascertain transcript co-localization, probes were detected with antibodies conjugated to horseradish peroxidase and were revealed by Tyramide Signal Amplification using Tyramide-FITC or -TAMRA as substrates. The specificity of the *uts2b* probe was verified using the sense *uts2b* probe as a negative control.

### Immunofluorescence

Whole-mount fluorescent immunohistochemistry was carried out on dissected and opened CNS (from stages 48 to 60) or on CNS sections (30 µm), after fixation in 4% paraformaldehyde (PFA) in phosphate-buffered saline (PBS) for 3 h at room temperature followed by rinsing three times for 10 min in PBS. Blocking of non-specific sites was performed for 2 h at room temperature in PBS, Triton X-100 0.3%, bovine serum albumin 1% (Sigma, St. Quentin Fallavier, France). Sampled were then incubated with the primary antibody diluted in the same buffer overnight at room temperature. They were then rinsed five times 10 min in PBS, incubated for 1h30 at room temperature with the fluorescently labeled secondary antibody, and washed again five times 10 min in PBS. The primary antibodies were goat anti-choline-acetyltransferase (ChAT; 1:100, Millipore) [46] and rabbit anti-GFP (1:300 dilution, Life technologies). The secondary antibodies were donkey anti-rabbit and anti-goat IgGs coupled to Alexa Fluor 488 and Alexa Fluor 546, respectively (1:500, Life Technologies). The specificity of the GFP antibody was verified on wild type animals, while the specificity of secondary antibodies was established by omitting the primary specific antibodies (data not shown).

α-bungarotoxin staining was performed on whole-mount stage 57 tadpoles fixed in 4% PFA for 1 h at room temperature according to Ymlahi-Ouazzani et al. [47]. Animals were incubated in Alexa 594-conjugated α-bungarotoxin (10 μg/ml; Life Technologies) overnight at 4°C, then rinsed six times 20 min in PBS, Triton X-100 0.1% at room temperature.

### Combined fluorescent *in situ* hybridization and immunofluorescence

*In situ* hybridization was performed before immunohistochemistry as described above and revealed using FITC- or TAMRA-conjugated tyramide. After several washes in PBS, tissues were submitted to immunochemistry as described above.

### Neuronal retrograde tracing

Animals were anesthetized in a 0.05% MS-222 water solution and transferred to a Sylgard-lined Petri dish to perform intramuscular dye injections. Spinal motoneurons were retrogradely labeled from various muscles at different critical developmental stages. Hereafter, motoneurons labeled from tail at stage 50 or 55 will be referred to as axial motoneurons, motoneurons labeled from dorsal trunk at stage 60 as thoracic dorsal motoneurons and motoneurons labeled from hindlimb buds at stage 50 and leg muscles at stage 55 or 60 as appendicular motoneurons [48–50]. First, the skin was dried before making a tiny incision to expose the muscles of interest. Crystals of fluorescent dextran amine dyes (Invitrogen) were applied intramuscularly with an insect pin. Either 3 kD rhodamine or 10 kD Alexa fluor 647 were used to be visually compatible with the GFP fluorescence wavelength in transgenic animals. Surplus dye was washed out with an excess of cold Ringer solution (75 mM NaCl, 25 mM NaHCO_3_, 2 mM CaCl_2_, 2 mM KCl, 0.5 mM MgCl_2_, and 11 mM glucose, pH 7.4). Tadpoles recovered from anesthesia in a water tank and were kept alive for a week to allow tracer migration into the motoneurons. Thereafter, spinal cords were dissected and fixed in 4% PFA for 12 h at 4°C. Neuronal retrograde tracing experiments combined with whole-mount fluorescent immunochemistry treatment (for GFP detection) were carried out as previously described in the immunofluorescence section. Preparations were incubated in a 20% sucrose solution (in PB 0.1%) for 24h at 4°C, then embedded in a tissue-tek solution (VWR-Chemicals) and frozen at −45°C in isopentane. 30 µm cross-sections were made using a Leica Cryostat.

### Electrophysiology

Electrophysiological recordings of spinal motor activity were made on isolated CNS preparations obtained from *uts2b*-GFP transgenic *X. laevis* tadpoles at stage 55–57. After anesthesia in 0.05% MS-222 the brainstem and the spinal cord were dissected out in a cold oxygenated Ringer solution (see composition above). Then, the isolated brainstem-spinal cord preparation was transferred to a Sylgard-lined recording chamber (volume ~2.5 mL) to be continuously superfused at a rate of ~2 mL/min in an oxygenated Ringer solution maintained at ~17°C. Spontaneous fictive locomotor activity was recorded from the 10–15^th^ ventral roots using borosilicate glass suction electrodes (tip diameter = 100 nm; Clark GC 120F; Harvard Apparatus), filled with the Ringer solution. The recorded signal was amplified (A-M system), rectified and integrated (time constant 100 ms; Neurolog System). Spinal GFP^+^ motoneurons were visualized within the whole-mount spinal cord (dorsal-side opened) using a differential interference contrast with an infrared video camera and a standard epifluorescent illumination system (FITC filter). Simultaneously to the spinal ventral root recordings, whole-cell patch-clamp recordings of GFP^+^ motoneurons were performed with a borosilicate glass patch-clamp electrode (pipette resistance = 5–6 MΩ; Clark GC 150TF; Harvard Apparatus) and filled with a solution containing 100 mM K-gluconate, 10 mM EGTA, 2 mM MgCl_2_, 3 mM Na_2_ATP, 0.5 mM NaGTP, 10 mM HEPES, pH 7.3. The intracellular signal was acquired using an Axoclamp 2A amplifier (Molecular Devices). All electrophysiological signals were computer-stored using a digitizer interface (Digidata 1440; Pclamp10 software; Molecular Devices) and analyzed off-line using the Clampfit software (Molecular Devices).

### Image acquisition

Samples stained by fluorescent probes and/or antibodies were acquired using laser scanning confocal microscopy (Zeiss LSM 510) at wavelengths of 405, 488, 543 and 633 nm. Stacks of 10 to 30 confocal images with 1–10 μm z-step intervals were generated with 10x/0.5 air and 20x/0.75 oil objectives. Final images presented in figures were obtained by orthogonal projection of entire stacks with artificial fluorescent colors using ZEN (Zeiss), Fiji [51] and Adobe Illustrator (Adobe Systems) softwares.

### Quantification of immunostained cells in wild type and transgenic tadpoles

Image stacks with 2–6 μm z-step intervals were performed from 72–120 μm-thick whole-mount dissected and opened spinal cords. Quantification of cells was carried out in a domain between the 12^th^ and 15^th^ segments that contained only axial motoneurons. To assess the *uts2b*/ChAT co-localization in wild type tadpoles, cells were counted in a region of 634 × 1000 μm width (n = 1). Note that the results obtained from this single specimen were globally consistent with those provided in three other ones. For GFP/*uts2b* and GFP/ChAT co-localizations in transgenic tadpoles, cells were counted in regions of 317 × 1500 μm width (n = 3, for each combination). Immuno-stained cells were manually counted in every plan of the z-stack by using the collaborative bioimage informatics platform, Icy [52].

## Results

### Expression pattern of the *uts2b* gene during development of wild type *X. laevis*

**Fig 2:**
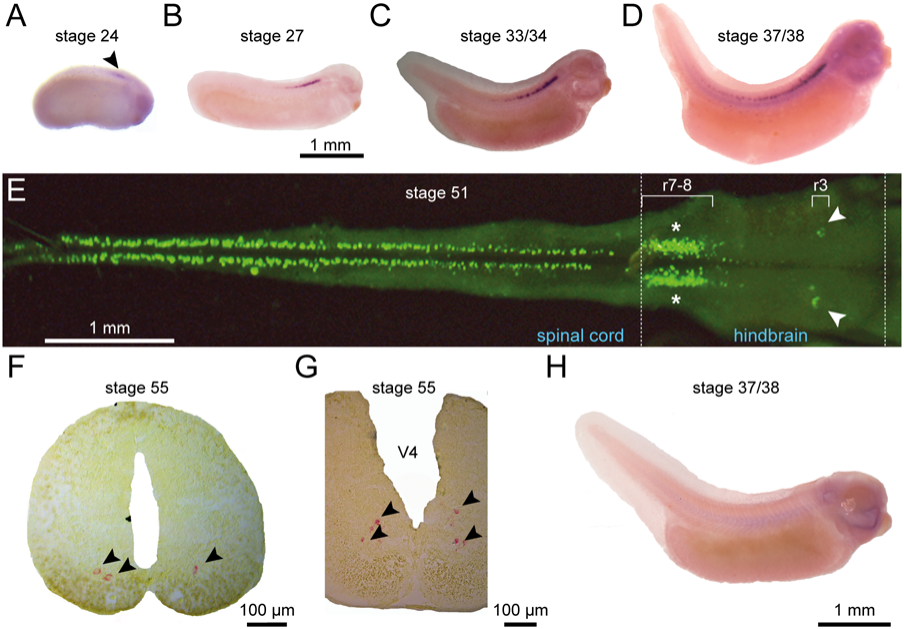
*uts2b* mRNA is restricted to the ventral spinal cord and hindbrainin *X. laevis* tadpoles. Distribution of the *uts2b* mRNA revealed by *in situ* hybridization with *uts2b* antisense probe on whole tadpoles (**A**–**D** and **H**), dissected and opened CNS (**E**), or CNS sections (**F**, **G**) at stages 24 (**A**), 27 (**B**), 33/34 (**C**), 37/38 (**D**) 51 (**E**), and 55 (**F**, **G**). **H**. Stage 37/38 tadpole hybridized with *uts2b* sense probe used as negative control. **A**–**D** and **H**. Lateral views of tadpoles with dorsal side up and rostral to the right. **E**. Dorsal view of whole-mount preparation of hindbrain-spinal cord (CNS). Note that in this preparation, the more ventral structures are closer to the midline. **F** and **G**. Coronal sections at the level of the spinal cord (**F**) and the posterior part of brainstem (**G**) with dorsal up. Arrowheads point to *uts2b* mRNA detection in stage 24 tadpole and in F and G coronal sections. White arrows designate the region of nuclei of the trigeminal (V) nerve; asterisks denote the region of nuclei of the vagus/hypoglossal/accessory (IX–XI) nerves. r, rhombomere; V4, fourth ventricule.

The constitutive expression of the *uts2b* gene in *X. laevis* was investigated by carrying out *in situ* hybridization on whole animals, dissected and opened CNS and CNS slices at a variety of different developmental stages (from stages 10 to 55 and in adults). The first *uts2b* signal could be detected in the rostral spinal cord from stage 24, pre-hatching early tailbud embryos (Fig. 2A). Gradually, the staining expanded rostro-caudally along almost the entire length of the spinal cord (Fig. 2B–E) and also appeared in the hindbrain (Fig. 2E). Cells with dense *uts2b* labeling were located in two parallel rows in the ventral part of the spinal cord (Fig. 2E, F). In the hindbrain, *uts2b^+^*cells were located in the same region as motor nuclei of the trigeminal nerve (V) in rhombomeric segments 2–3 and vagus/hypoglossal/accessory nerves (IX-XI) in rhombomeric segments 7–8, respectively (Fig. 2E,G) [53]. A few *uts2b^+^* cells were still observable in the spinal cord of a small proportion of stage 66 juveniles (data not shown), but no longer in adults, whereas the *uts2b* gene was still strongly expressed in the hindbrain during adulthood (data not shown). No hybridization signal was observed with the sense *uts2b* riboprobe (Fig. 2H).

**Fig 3:**
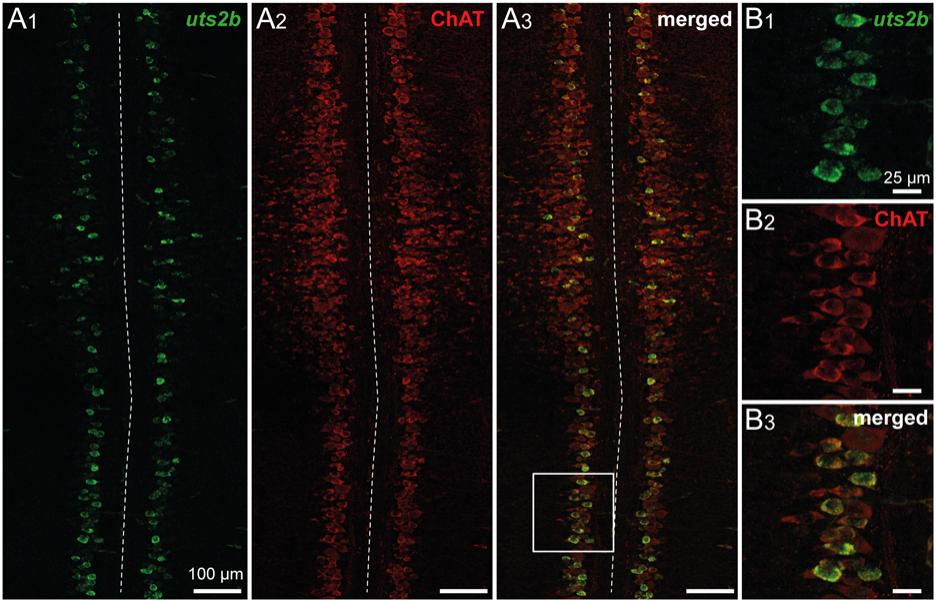
Spinal *uts2b* ^+^ cells are cholinergic neurons in *X. laevis* tadpoles. Confocal images of combined fluorescent in situ hybridization of uts2b mRNA (**A1**, **B1**) and ChAT immunolabeling (**A2**, **B2**) in a whole-mount dissected and opened spinal cord preparation of stage 50 wild type tadpole. **A3**, **B3**. Merged image obtained when uts2b and ChAT stainings were superimposed. Dorsal view with rostral up. Note that in this preparation, the more ventral structures are close to the midline (dashed line). The boxed region in **A3** is shown at higher magnification in **B1**–**B3**.

To better characterize the *uts2b^+^* cells in the spinal cord, we performed single fluorescent *in situ* hybridization using the *uts2b* probe followed by immunohistochemistry labeling against ChAT, a marker of cholinergic neurons. As depicted in Fig. 3A, *uts2b^+^* cells were restricted to the ventro-medial part of the cholinergic area in the spinal cord of stage-50 tadpoles and they were all found to express ChAT (Fig. 3B), in agreement with their putative motoneuronal nature. *uts2b* mRNA was visualized in a little more than half of all the ChAT^+^ cells analyzed.

**Fig 4:**
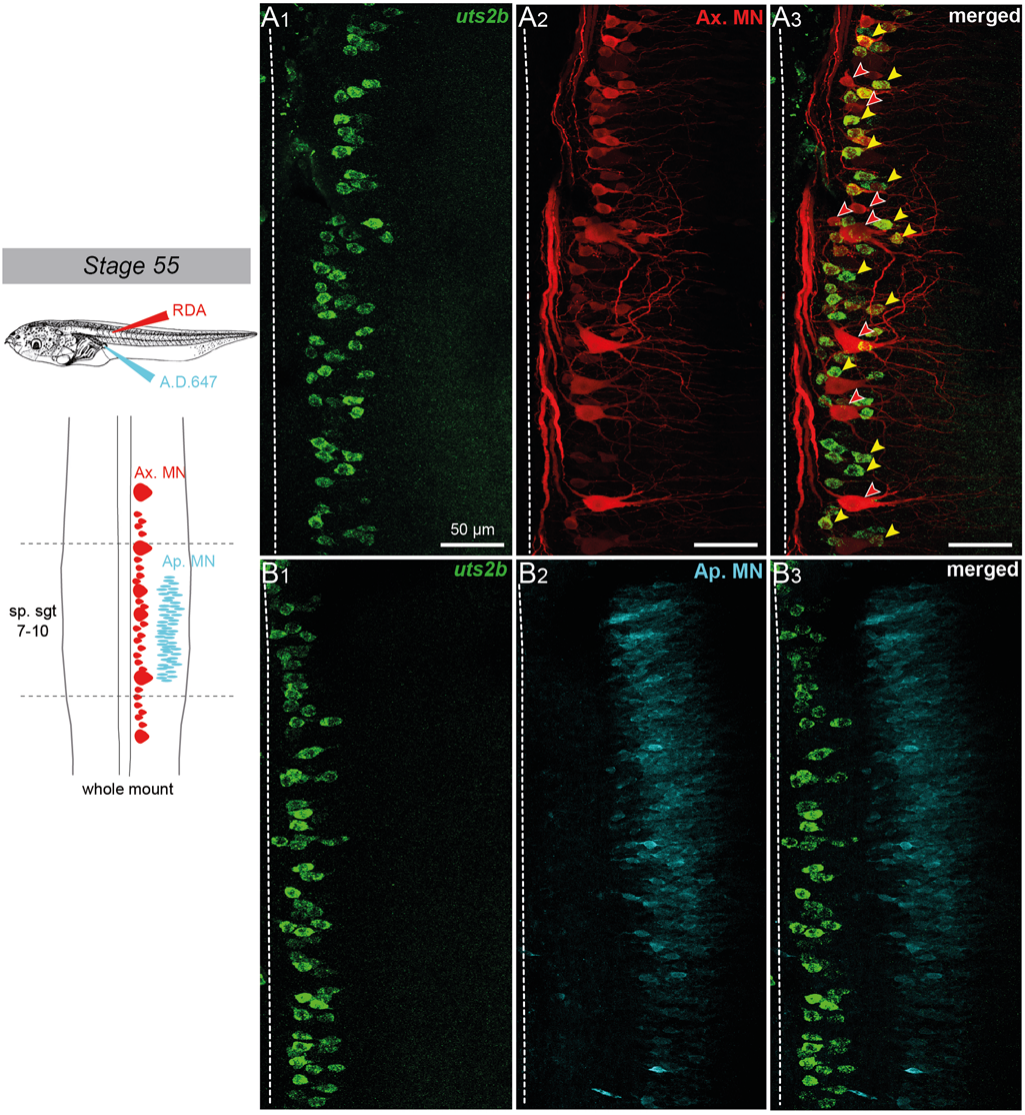
Spinal *uts2b* ^+^ cells of *X. laevis* tadpoles project to tail myotomes. Confocal image of combined fluorescent *in situ* hybridization of *uts2b* mRNA (**A1**, **B1**) and retrograde labeling of spinal axial (**A2**) and appendicular (**B2**) motoneurons in stage 55 tadpole whole-mount spinal cord preparations. **A3**, **B3**. Merged images of *uts2b* staining and retrograde labeling. All images display dorsal view of hemi-cords, with the rostral side up (dashed-line on the left indicates the midline). Axial motoneurons (Ax MN) were labeled with rhodamine dextran dye (RDA) injected into tail myotomes while appendicular motoneurons (Ap MN) were labeled from posterior leg muscles with Alexa Dextran 647 dye (A.D. 647; see upper scheme on the left panel). The drawing on the left panel illustrates the localization of Ax MN and developing Ap MN in larval spinal cord at stage 55. Yellow arrowheads indicate double stained cells. Red arrowheads indicate *uts2b—*retrograde labeled cells. sp.sgt 7–10, spinal segments 7 to 10.

Selective retrograde motoneuron labeling was performed with intramuscular application of fluorescent dextran amine dyes, in order to unravel which motoneuron pools expressed *uts2b*. Axial motoneurons were labeled from the first 10–12^th^ tail myotomes (Fig. 4A) whereas appendicular motoneurons were labeled from posterior leg *gluteus magnus* and *semimenbranosus* muscles (Fig. 4B), both in stage 55 tadpoles. A significant proportion of the axial retrogradely labeled motoneurons appeared to contain *utsb* mRNA (yellow arrowheads; Fig. 4A3). In contrast, none of the retrogradely labeled appendicular motoneurons expressed *utsb* (Fig. 4B). Note that some axial motoneurons retrogradely labeled did not contain *uts2b* mRNA (red arrowheads; Fig. 4A3) suggesting that *uts2b* is expressed in only a subset of these neurons.

### Generation of *uts2b*-GFP transgenic *X. laevis* tadpoles

**Fig 5:**
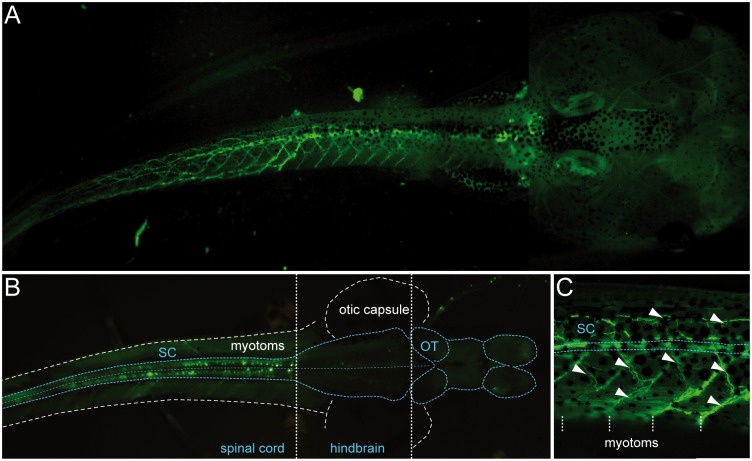
Most of the fluorescence visible in transgenic *uts2b*-GFP *X. laevis* tadpoles occurs in cells located in the spinal cord and in motor axon projections. **A**. GFP fluorescence imaging of a representative transgenic tadpole at stage 58. The tail is seen in lateral view while the head is seen in dorsal view. **B**. GFP expression at the level of a dissected and opened whole-mount CNS of a stage 50 transgenic tadpole. The CNS was optically exposed by removing the dorsal part of the tail and the top of the head. Note that GFP^+^ cells are restricted to the spinal cord. Dorsal view, rostral to the right. **C**. Detail of **A** showing GFP^+^ motor axon projections (arrowheads) extending towards axial musculature. White dashed vertical lines indicate myotome boundaries. SC, spinal cord. OT, optic tectum.

Since we found that *uts2b* was constitutively active in cells that correspond to spinal motoneurons, *uts2b* promoter was a good candidate to use to drive the expression of GFP in transgenic *Xenopus* motoneurons. To generate *uts2b*-GFP transgenic tadpoles, we used a BAC clone that carried the whole *uts2b* genomic region including the upstream and downstream regions. GFP cDNA was substituted in the first exon of the coding sequence of the uts2b gene, in the BAC clone (process steps summarized in Fig. 1). To test the recombineered *uts2b*-GFP BAC for expression in transgenic animals, circular recombineered BAC DNA was injected into one-cell stage *X. laevis* embryos, as previously described by Fish et al. (2012). 24.9% of the *uts2b*-GFP transgenic tadpoles (out of the 2061 injected embryos that were still alive at stage 40 in total) exhibited the expected GFP expression pattern with a robust, extensive rostro-caudal distribution of spinal GFP^+^ cells (Fig. 5A) with labeling of soma (Fig. 5B) and neuronal ramifications (Fig. 5A,C). 18.4% of transgenic tadpoles showed a reduced GFP expression profile, restricted to the soma or to a smaller part of the spinal cord. In contrast, 3.3% showed only ectopic expression of GFP while 53.3% were totally devoid of fluorescence. It is worth noting that in a number of the transgenic tadpoles, the fluorescence was expressed in just one lateral half of the body (data not shown).The GFP expression was seen in spinal neurons without ambiguity, namely both in their somata and axonal projections, from stage 40, until at least stage 62–63. However, the first fluorescence signal could be detected as early as 32, but only as small dots in the rostral spinal cord. It is noteworthy that in some animals, spinal GFP^+^ cells could be detected until stages 65–66. Due to their ventro-medial distribution in the spinal cord (Fig. 5) and their typical morphology (*i.e.* multipolar soma with ventral and dorsal dendrites and an axon projecting ventrally and caudally toward the axial musculature) it is likely that the GFP^+^ cells of the *uts2b*-GFP transgenic tadpoles were spinal motoneurons, as compared to the previously described *X. laevis* tadpole motoneurons [49, 54]. Surprisingly, no GFP^+^ cells were detected in the hindbrain (Fig. 5B), in contrast to the endogenous *uts2b* (Fig. 2E, G).

**Fig 6:**
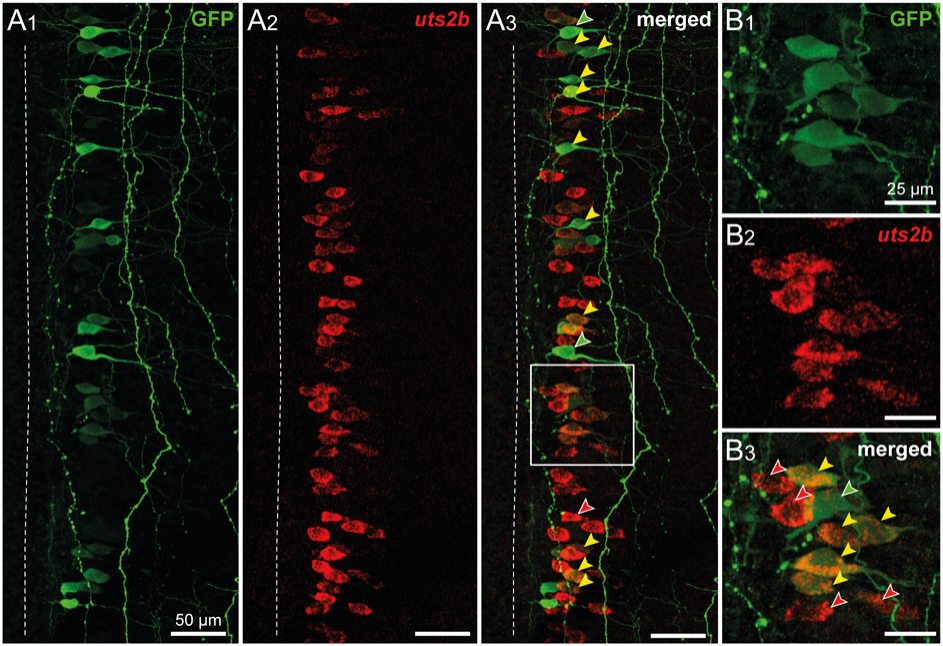
The *uts2b* expression pattern is partially reproduced by GFP in transgenic *uts2b*-GFP *X. laevis* tadpoles. Confocal images of combined immuno-labeling of GFP^+^ neurons (**A1**, **B1**) and fluorescent *in situ* hybridization of *uts2b* mRNA (**A2**, **B2**) in a whole-mount spinal cord preparation of stage 50 *uts2b*-GFP tadpole. **A3**, **B3**. Merged image obtained when GFP and *uts2b* stainings were superimposed. Dorsal view with rostral up. Only the hemi-cord is shown. The dashed-line on the left side represents the midline. Note that in this preparation, the more ventral structures are closer to the midline. The boxed region in **A3** is shown at higher magnification in **B1**–**B3**. Yellow arrowheads denote cells that express both GFP and *uts2b*. Red arrowheads designate *uts2b* ^+^/GFP^−^ cells and green arrowheads designate *uts2b* ^−^/GFP^+^ cells.

To further compare the GFP and endogenous *uts2b* expression patterns, we performed an *in situ* hybridization using the *uts2b* antisense probe combined with an anti-GFP immunostaining in the stage 50 *uts2b-*GFP transgenic tadpoles. 82.0 ± 7% of the spinal GFP^+^ cells (n = 3 animals, with ~ 85 GFP^+^ cells counted per animal) were also *uts2b^+^* (yellow arrowheads; Fig. 6A3, B3). However, 46 ± 9% of the *uts2b* ^+^ cells (n = 3 animals, with ~ 160 *uts2b^+^* cells counted per animal) did not contain GFP (red arrowheads; Fig. 6A3, B3).

### Neurochemical and neuroanatomical characterization of the GFP^+^ cells in transgenic *X. laevis* tadpoles

**Fig 7:**
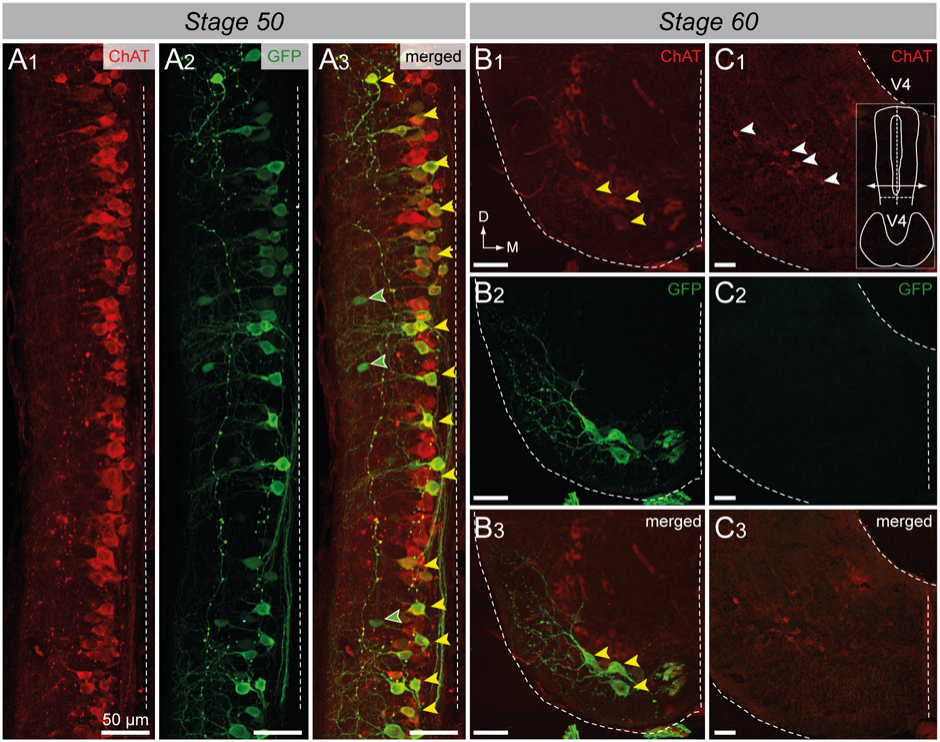
Spinal GFP^+^ cells of transgenic *uts2b*-GFP *X. laevis* tadpoles mainly express ChAT. Confocal images of combined immuno-labeling of ChAT^+^ (**A1**) and GFP^+^ (**A2**) neurons in a whole-mount spinal cord preparation of stage 50 transgenic tadpole. **A3**. Merged image obtained when ChAT and GFP stainings were superimposed. Dorsal view with rostral up. Only the hemi-cord is shown. The dashed-line on the right side represents the midline. Note that in this preparation, the more ventral structures are closer to the midline. Confocal images of combined immuno-labeling of ChAT^+^ (**B1**–**C1**) and GFP^+^ (**B2**–**C2**) neurons in lumbar spinal cord (**B**) and brainstem (**C**) cross-sections of stage 60 *uts2b*-GFP tadpoles. **B3**, **C3**. Merged images obtained when ChAT and GFP stainings were superimposed. The cross-section in **C1**–**3** originates from the caudal hindbrain (rhombomeres 7–8) where IX-XI motor nuclei are located. Yellow arrowheads designate GFP^+^/ChAT^+^ cells; green arrowheads designate GFP^+^/ChAT^−^ cells; White arrowheads designate GFP^−^/ChAT^+^ cells. D, dorsal; M, medial; V4, 4^th^ ventricule.

In order to examine the putative motoneuronal nature of GFP^+^ spinal neurons, co-localization of ChAT immuno-fluorescence was investigated in *uts2b*-GFP transgenic tadpoles exhibiting a strong fluorescence both in the spinal somata and axon projections (Fig. 7). Double immuno-labeling against GFP and ChAT demonstrated that almost all spinal GFP^+^ neurons expressed ChAT at all developmental stages tested. Indeed, at stage 50, 98.2 ± 0.5% (n = 3 animals, ~140 GFP^+^ cells counted per animal) of the GFP^+^ cells were ChAT^+^. Conversely, 45.0 ± 5.4% (n = 3 animals, ~300 ChAT^+^ cells counted per animal) of the ChAT^+^ neurons were also GFP^+^ (yellow arrowheads; Fig. 7A3), indicating that the *uts2b*-driven GFP expression was restrained to a specific subpopulation of cholinergic neurons, as reported earlier for endogenous *uts2b* expression. At stage 60, all spinal ventro-medial GFP^+^ neurons were also found to express ChAT (yellow arrowheads; Fig. 7B3). Here too, we did not observe GFP^+^ cells in the hindbrain (Fig. 7C).

**Fig 8:**
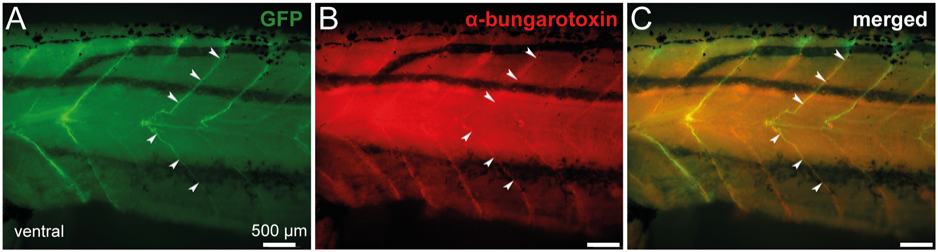
Spinal GFP^+^ axon motor projections of transgenic *uts2b*-GFP *X. laevis* tadpoles co-localize with α-bungarotoxin, a marker of postsynaptic neuromuscular junctions. Combined fluorescence of GFP (**A**) and immuno-labeling of α-bungarotoxin (**B**) at the level of spinal axon motor projections in a whole-mount stage 57 transgenic tadpole. **C**. Merged image obtained when GFP fluorescence and α-bungarotoxin staining were superimposed. Postsynaptic nicotinic acetylcholine receptors labeled by α-bungarotoxin and GFP^+^ motor neuron axons overlap (arrowheads). Lateral views, rostral to the left.

Axonal projections of spinal GFP^+^ cells were further characterized in stage 57 transgenic tadpoles, using α-bungarotoxin staining to locate the neuromuscular junctions in the axial musculature. As shown in Fig. 8, α-bungarotoxin fluorescence revealed clusters of nicotinic acetylcholine receptors (Fig. 8B) that were co-localized with GFP^+^ axon terminals (Fig. 8C). Such a specific neuromuscular arrangement was, again, supportive of a motoneuronal nature for GFP^+^ neurons.

**Fig 9:**
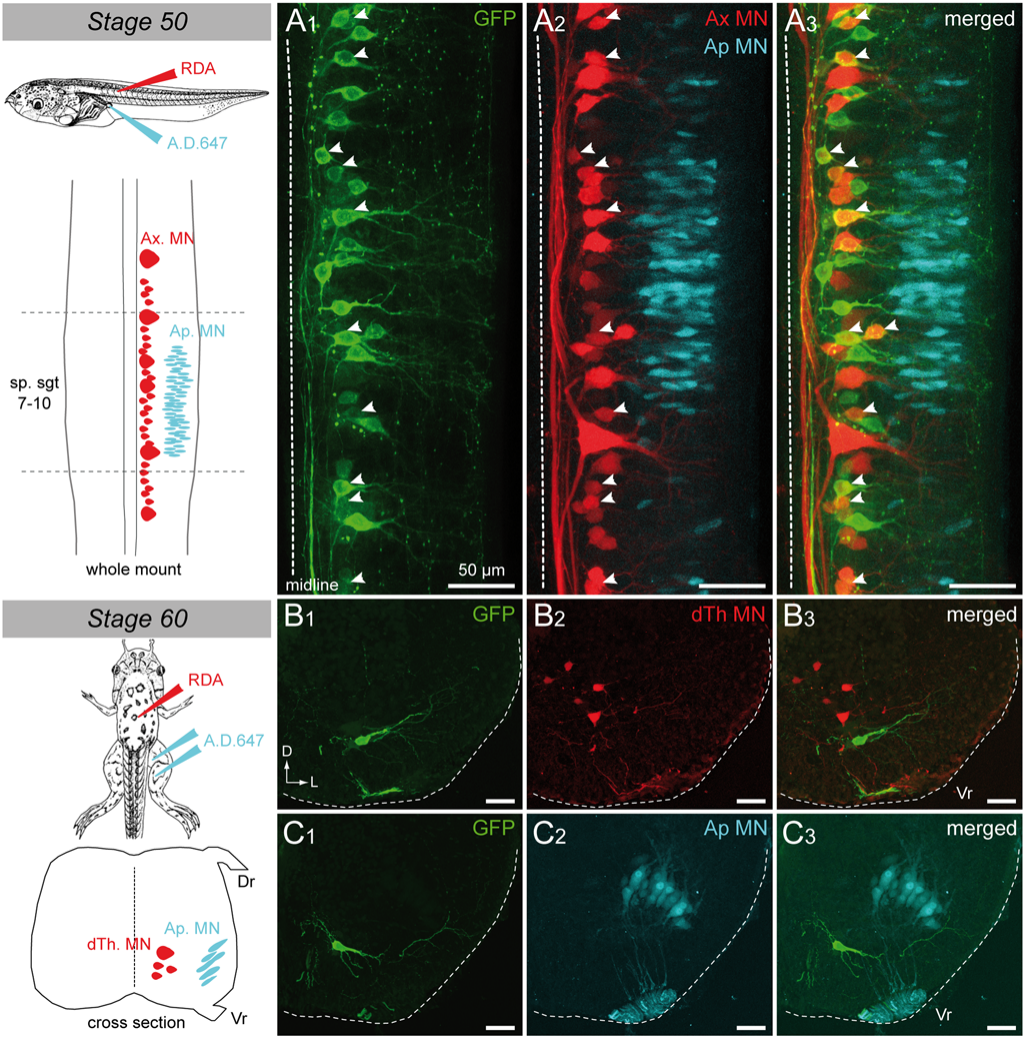
Spinal GFP^+^ cells of transgenic *uts2b*-GFP *X. laevis* tadpoles project to tail myotomes. Confocal image of combined immuno-labeling of spinal GFP^+^ neurons (**A1**) and retrograde labeling of spinal motoneurons (**A2**) in a stage 50 *uts2b*-GFP tadpole whole-mount spinal cord preparation. **A3**. Merged image obtained when GFP staining and retrograde labeling were superimposed. Dorsal view with rostral up. Only the hemi-cord is shown. The dashed-line on the right side represents the midline. Axial motoneurons (Ax MN) innervating myotomes were labeled from tail muscles with rhodamine (RDA) dextran dye whereas appendicular motoneurons (Ap MN) innervating limbs were labeled from hindlimb buds with alexa dextran 647 dye (A.D. 647; see scheme on the left panel). The drawing at the top left panel illustrates the medio-lateral localization of Ax MN and developing Ap MN in larval spinal cord at stage 50. Yellow arrowheads indicate double stained cells. Cross-section confocal image of combined immuno-labeling of spinal GFP^+^ neurons (**B1**, **C1**) and retrograde labeling of dorsal thoracic motoneurons (dTh MN, **B2**) and Ap MN, (**C2**) in stage 60 metamorphosing *uts2b*-GFP tadpole. **B3**, **C3**. Merged images obtained GFP staining and retrograde labeling were superimposed. dTh MN were labeled from *dorsalis trunci* muscles with RDA whereas Ap MN were labeled from groups of extensor and flexor of the hindlimb with A.D. 647 (see scheme on the left side). The cross-section drawing at the bottom left panel illustrates the dorso-ventral localization of dTh MN and Ap MN in the future adult spinal cord at stage 60. sp.sgt 7–10, spinal segments 7 to 10, Vr, ventral root. D, dorsal; L, lateral.

Selective retrograde motoneuron labeling was performed as previously described in wild-type specimens in order to unravel which motoneuron pools expressed GFP from pre- to post-metamorphic *uts2b*-GFP transgenic *Xenopus*. Axial motoneurons were labeled from the first 10–12^th^ tail myotomes at stage 50 (Fig. 9A); thoracic dorsal motoneurons were labeled from *dorsalis trunci* muscles at stage 60 (Fig. 9B) whereas appendicular motoneurons were labeled from hindlimb buds at stage 50 (Fig. 9A) and from the *gluteus magnus* and *semimenbranosus* leg muscles at stage 60 (Fig. 9C). A significant proportion of the axial motoneurons retrogradely labeled appeared to be GFP^+^ (white arrowheads; Fig. 9A). This demonstrated that the *uts2b*-GFP transgene was expressed in tail myotome-innervating motoneurons in pre-metamorphic stages 50–58, corroborating the above ChAT immuno-labeling patterns (Fig. 7). In contrast, none of the retrogradely labeled thoracic dorsal (Fig. 9B) or appendicular (Fig. 9A, C) motoneurons expressed GFP.

### Electrophysiological characterization of the GFP^+^ cells in transgenic *X. laevis* tadpoles

**Fig 10:**
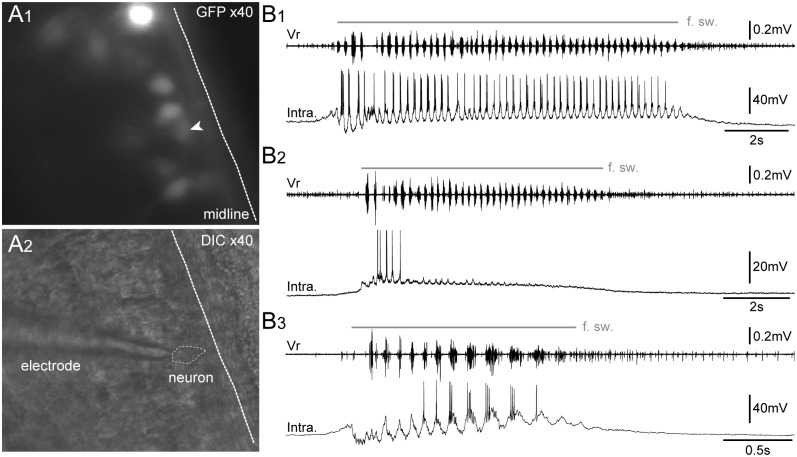
GFP^+^ cells exhibit a typical pattern of motoneuronal electrical activity during spontaneous fictive swimming episodes. Visualization of spinal GFP^+^ neurons at x40 in fluorescence condition (**A1**) and infrared condition (**A2**) in a whole-mount isolated *in vitro* preparation of brainstem-spinal cord with dorsal-side opened. Patch-clamp intracellular recordings (bottom traces) of spinal GFP^+^ neurons (Intra.) either with a persisting (**B1**), a non-persisting firing pattern (**B2**), or a slow-delayed firing pattern (**B3**) during spontaneous fictive swimming episodes (f. sw.). The upper trace in **B1**–**B3** represents the extracellular fictive swimming activity recorded simultaneously in a ventral motor root (Vr). DIC, differential interference contrast. The white arrowhead points to the GFP^+^ neuron targeted for patch clamp recording.

GFP^+^ spinal neurons were specifically targeted for patch-clamp recordings (Fig. 10A) in order to functionally characterize their electrophysiological activity during the spontaneous fictive swimming (Fig. 10B) in *in vitro* isolated spinal cord preparations. All recorded GFP^+^ neurons (n = 17) were rhythmically active in coordination with the fictive swimming bursts of activity recorded from axial ventral motor roots (Vr). Three main intracellular discharge patterns were observed: *i)* some neurons (n = 5) were depolarized at the onset of the fictive swimming and fired rhythmically coupled with Vr bursts throughout the episode duration (Fig. 10B1); *ii)* some neurons (n = 4) stopped firing after the 3–4 first Vr bursts despite the persistence of the swimming activity, however, these neurons continued to exhibit rhythmic depolarizations in strict coordination with the following Vr bursts (Fig. 10B2); *iii)* the other neurons (n = 8) were hyperpolarized at the beginning of the fictive swimming episode and started to fire in coordination with the Vr rhythmic bursts with a delay after the swimming onset (Fig. 10B3). For all recorded GFP^+^ neurons, the membrane potential was between ‑53 and ‑68 mV. These electrophysiological results are consistent with locomotor-related firing patterns previously described in motoneurons of larval *Xenopus* [55] and zebrafish [56–58].

Taken together, neurochemical, anatomical and electrophysiological results demonstrate that GFP^+^ spinal neurons represent a heterogeneous subpopulation of axial motoneurons innervating tail muscles.

## Discussion

The aim of this study was to test the ability of the regulatory sequences of the *uts2b* gene to drive expression of reporter genes in *Xenopus* motoneurons. Expression of *uts2b* in motoneurons of the brainstem and spinal cord has been formally demonstrated in several species including the mice [28], chicken and zebrafish (HT, unpublished results). In mice, it has been shown that a majority of spinal motoneurons simultaneously expresses *uts2b* and its paralog *uts2* [27]. In *X. laevis*, Konno et al. [29] reported the occurrence of UTS2/UTS2B-immunoreactive motoneurons in the brain and spinal cord but due to the technique employed they were not able to discriminate the cells expressing specifically each peptide. Here, we provide evidence that in pre-metamorphic tadpole, spinal *uts2b^+^* cells are motoneurons, as revealed by the fact that they express ChAT and can be retrolabeled from axial muscles. The onset of the *uts2b* gene expression (stage 24), which appears shortly after differentiation of the first motoneurons (up to stage 22) [59], is consistent with this view, even if at earliest stages, the motoneuronal nature of the *uts2b^+^* cells remains to be confirmed. At later stages, *uts2b* mRNA was detected in retrolabled axial motoneurons but not with appendicular motoneurons suggesting that *uts2b* is specifically expressed in axial motoneurons innervating tail muscles.

The absence of *uts2b* ^+^ cells in the spinal cord of adult specimens was quite surprising since *uts2b* mRNA in spinal cord was detected by RT-PCR [29]. Our results indicate that *uts2b* expression pattern of *X. laevis* in adult is very similar to that found in rat. Indeed, the rat spinal cord has been shown to contain only *uts2* but not *uts2b* mRNA [25].

The *X. laevis uts2b* expression pattern suggests the presence, in its promoter, of regulatory elements that are able to drive motoneuron-specific expression. Since BAC technology makes it possible to generate transgenes even when the promoter region of a gene is unknown, which was the case here, we decided to generate transgenic tadpoles expressing the EGFP reporter from a BAC containing the entire *X. tropicalis uts2b* gene including more than 130 kbp of upstream and almost 20 kbp of downstream regulatory elements. BAC transgenesis has been successfully used for little over a decade in mouse [38], and recently has been more readily applied to other species such as zebrafish [60]. In *Xenopus*, trials of BAC transgenesis have been reported in only two studies so far [35, 61]. We opted for the method developed by Fish et al. [35] due to its simplicity and high efficiency. Up to 25% of the *uts2b*-GFP transgenic tadpoles globally exhibited the expected pattern of fluorescence in our case, admittedly somewhat lower than that reported in the original publication (*i.e.* 60%) but nonetheless a very acceptable rate. The delay between the first *uts2b* signal (stage 24) and the first GFP fluorescence signal (stage 32) may correspond to the time needed to complete both translation and then maturation of GFP [62].

Some other discrepancies were nevertheless observed between the GFP and endogenous *uts2b* expression patterns. No fluorescence was expressed in the hindbrain, suggesting that some of the regulatory elements of the *X. tropicalis uts2b* gene, crucial for its expression in the brainstem, were not present in the BAC, or not functional in *X. laevis* cells. At the spinal cord level, many *uts2b* ^+^ cells expressed GFP. However, we also observed a number of *uts2b* ^+^ cells that did not express GFP, as well as some GFP^+^ cells that did not express *uts2b*. The *uts2b* ^+^ /GFP- cells could be cells lacking the *uts2b*-GFP BAC DNA, or containing only a very small number of copies. Alternatively, these *uts2b* ^+^ /GFP^−^ cells could represent newly differentiated neurons in which GFP had not the time to accumulate, since motoneurogenesis continues to be active during a large part of the larval period (up to stage 52) [63]. The occurrence of GFP^+^/*uts2b-* cells might potentially reflect temporal variations in *uts2b* expression in motoneurons, *i.e.* motoneurons that had temporarily ceased to express *uts2b* but still contained GFP. However, the fact that all spinal GFP^+^ cells of our *uts2b*-GFP transgenic tadpoles were motoneurons is supported by several lines of evidence: *i)* they were exclusively located in the ventral horns of the spinal cord; *ii)* they were almost all ChAT^+^; *iii)* they could be stained by retrograde labeling from axial muscles; *iv)* they exhibited locomotion-related rhythmic discharges during spontaneous fictive swimming episodes and *v)* the axon terminals co-localized with α-bungarotoxin. However, as mentioned previously for *uts2b* in wild-type, only a fraction of the motoneurons appeared to express GFP. Retrograde labeling reveals that these neurons correspond to axial motoneurons, which innervate tail muscles, but not to appendicular or thoracic motoneurons. In this respect, the GFP expression pattern is perfectly consistent with endogenous *uts2b* expression in spinal cord of wild-type tadpoles. The fact that very few spinal GFP^+^ cells could be identified in early post-metamorphic juveniles also supports this view, since axial motoneurons elimination starts during metamorphosis as the tail regresses [64–66]. The higher number of spinal GFP^+^ cells in transgenic juveniles compared to that of *uts2b^+^* cells in wild-type ones is probably due to the higher stability of EGFP [67].

We used a transgenesis method in which the BAC DNA is injected into one-cell stage embryos as a circular DNA molecule with no mechanism to mediate genomic integration [35]. It is therefore believed that the injected DNA can be only transiently transcribed unless it is replicated in a similar fashion as the nuclear DNA. Since BAC DNA can contain a large amount of genomic DNA, it is more efficiently replicated [35]. In their original study using a *pax6*-GFP BAC, Fish et al. [35] actually reported loss of transgene expression around stage 50 in most of the injected embryos. In the present study, we observed that in most transgenic tadpoles, GFP was still significantly expressed up until stage 62–63 and, as mentioned above, GFP^+^ cells could be even observed in stage 66 juveniles. Thus, the decrease in GFP expression observed during metamorphosis may not be due to any BAC loss but, rather, reflects the endogenous expression pattern of *uts2b*. Maintenance of the transgene expression for such a long time may be explained by BAC DNA episomal replication and a particular stability of the intracellular environment, perhaps related to the post-mitotic state of motoneurons [68].

## Conclusion

In this study, we successfully demonstrated that *uts2b* is an appropriate driver to express reporter genes in a particular subpopulation of *Xenopus* tadpole spinal motoneurons. The transgenic *uts2b*-GFP tadpoles we generated constitute a potentially valuable model for further studying the spinal motoneurons, both in the physiological and pathological context, and for screening candidate therapeutic molecules modulating motoneuron survival, although the latter would require further development of stable transgenic lines. In the meantime, transient transgenesis using the *uts2b*-GFP BAC could represent an excellent tool to identify regulatory elements involved in the motoneuronal expression of the *uts2b* gene.

## Supporting Information

S1 Table Sequences of the primers used for PCR amplifications. (DOCX)
