# Supplementary material for: Generation of BAC Transgenic Tadpoles Enabling Live Imaging of Motoneurons by Using the Urotensin II-Related Peptide (ust2b) Gene as a Driver
Source: PLoS One. 2015 Feb 6;10(2):e0117370. doi: 10.1371/journal.pone.0117370 (PMC4319907; doi:10.1371/journal.pone.0117370)
Supplement: S1 Table — (DOCX) [file pone.0117370.s001.docx]

| Recombineering  *(N.B. Upper cases indicate the sequences homologous to the BAC).* | | |
| --- | --- | --- |
| *utsd* 5’ | *utsd* 5’arm_S | 5’-ccgaagcTTGTGACACCTCTGAAGACC-3’ |
|  | *utsd* 5’arm_AS | 5’-tatCCATggTATTGGCTCTTCAGCTGATGC-3’ |
| *utsd* 3’ | *utsd* 3’arm_S | 5’-ttcccatgGCTTGTTTGCAAATTGAGCTAG-3’ |
|  | *uts*d 3’arm_AS | 5’-aaagcggccGCACTCTGTATAAACAGACCTG-3’ |
| *uts2b* InFusion | InFusion_S | 5’-agagccaataccatgAGCAAGGGCGAGGAGCTG-3’ |
|  | InFusion_AS | 5’-tgcaaacaagccatgGCAGTCGACGGTGCAGG-3’ |
| *ccdc50* 5’ | *ccdc50* 5’arm_S | 5’-GCTAGAATTCCTGCCTAACTTTTACACGC-3’ |
|  | *ccdc50* 5’arm_AS | 5’- tttggaTCCCGGTAGCTTAGACTGATC-3’ |
| *ccdc50* 3’ | *ccdc 50* 3’arm_S | 5’- aacggaTCCAACTCTGTGCGCAGG-3’ |
|  | *ccdc 50* 3’arm_AS | 5’- caatcTAGATGTGCCAACGTGCAGTAAAG-3’ |
| Probe synthesis | | |
| *uts2b* | *Uts2b*_S | 5’-GGGGCAGTTAAAGCCTC-3’ |
|  | *Uts2b*_AS | 5’-AAATGAAACGGTGCTTTTGC-3’ |
